# *In vitro* electrochemical assessment of electrodes for neurostimulation in roach biobots

**DOI:** 10.1371/journal.pone.0203880

**Published:** 2018-10-10

**Authors:** Tahmid Latif, Michael McKnight, Michael D. Dickey, Alper Bozkurt

**Affiliations:** 1 Department of Electrical and Computer Engineering, North Carolina State University, Raleigh, NC, United States of America; 2 Department of Chemical and Biomolecular Engineering, North Carolina State University, Raleigh, NC, United States of America; US Naval Research Laboratory, UNITED STATES

## Abstract

Biobotics investigates the use of live insects as biological robots whose locomotion can be controlled by neurostimulation through implanted electrodes. Inactivity in the biobots (*bio*logical ro*bots*) can sometimes be noticed following extended neurostimulation, partly owing to incompatibility of implanted electrodes with the biobotic application or gradual degradation of the tissue-electrode interface. Implanted electrodes need to sufficiently exhibit consistent, reliable, and stable performance during stimulation experiments, have low tissue-electrode impedance, facilitate good charge injection capacity, and be compact in size or shape. Towards the goal of finding such electrodes suitable for biobotic applications, we compare electrochemical performances of five different types of electrodes *in vitro* with a saline based electrolytic medium. These include stainless steel wire electrodes, microfabricated flexible gold electrodes coated with PEDOT:PSS conductive polymer, eutectic gallium indium (EGaIn) in a tube, and “hybrid” stainless steel electrodes coated with EGaIn. We also performed accelerated aging of the electrodes to help estimate their longitudinal performance. Based on our experimentation, microfabricated electrodes with PEDOT:PSS and stainless steel electrodes coated with EGaIn performed remarkably well. This is the first time conductive polymer and liquid metal electrodes were studied comparatively for neurostimulation applications. These *in vitro* comparison results will be used in the future to provide a benchmark for subsequent *in vivo* tests with implanted electrodes in cockroach biobots.

## Introduction

**Fig 1:**
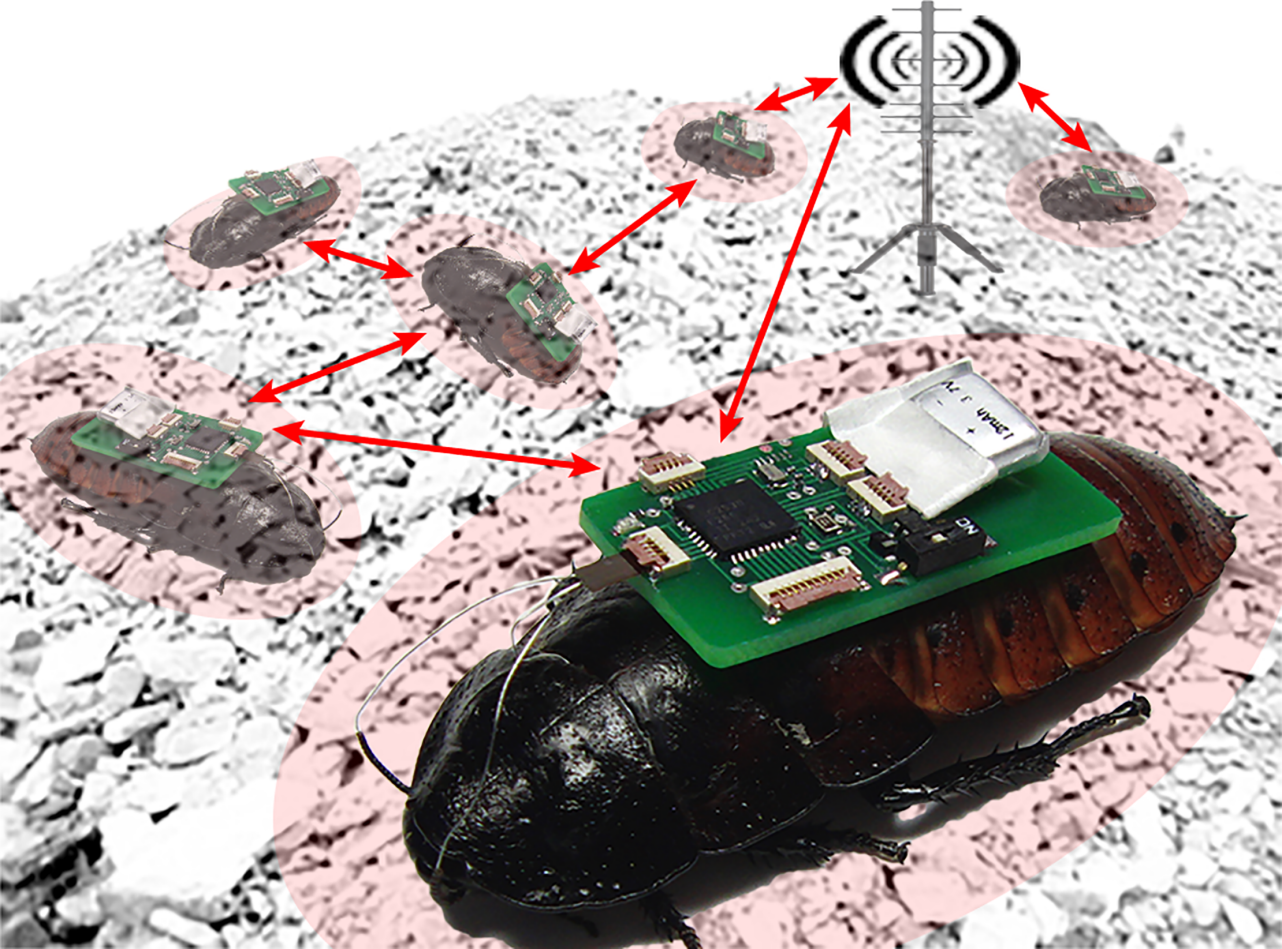
Illustration of a cyber-physically organized under-rubble biobotic swarm based mobile sensor network. The illustration uses real images of insect biobots with neurostimulation backpacks and implanted stainless steel wire electrodes. Each of the ZigBee enabled system-on-chip based backpacks can be configured as a sensor node in the network.

Electrodes implanted in certain locations of an insect’s body can be used for applying the required muscular or neural stimulation to evoke predetermined effects. System-on-chip-based electronic backpacks have been proven successful in controlling direction of locomotion in both aerial [1,2] and terrestrial [3–5] biobotic insects. Flight muscle stimulation in moths evokes wing flapping [1] while antennal neurostimulation in cockroaches leads to changing direction of motion via obstacle simulation [3,6]. We applied unilateral voltage-controlled stimuli at the antennae of Madagascar hissing cockroaches (*Gromphadorhina portentosa*) to make the roach biobots (*bio*logical ro*bots*) or roach-bots turn in the opposite direction of a stimulus; they turn left when the right antennae is stimulated and vice versa to navigate through mazes and autonomously locate sound sources [3,7].These biobots are envisioned as “working” insects to carry sensors in emergency response environments to sites that are beyond the reach of dogs or conventional robots [3,8]. First responders in search and rescue missions after natural disasters, in particular, can benefit from the use of a cyber-physically organized swarm of these biobots to search the deeper parts of the rubble (Fig 1). In this regard, reliability on these biobots is of the utmost concern.

Laboratory-based experiments with our roach biobots usually run in the range of 2–3 hours a day spanning over a week. With progression of time, a gradual decrease or inactivity has sometimes been observed in biobotic response to neurostimulation. Neurostimulation in biobots indeed derives its use from a wide clinical application base where such deterioration of electrode performance over time has been a major issue [9]. In brain-machine interface studies, the advent of microfabricated electrode arrays has enabled more reliable methods of neural stimulation and recording in peripheral and central nervous systems. While acute recording and stimulation experiments have proven to be sound, reliability and stability become an issue for chronic experiments running over a longer period of time [9,10]. Reasons are often not clear, but has been cited as due to changes in the tissue-electrode interface caused by the electrode shifting, fibrous encapsulation, biocompatibility issues, and electrode corrosion [9,10]. Such activities would also be the cause of failing bioelectrical coupling between the biobotic insect tissue and electrodes [11,12].

Electrochemical assessment techniques like electrochemical impedance spectroscopy can provide quantitative information on the quality of the tissue-electrode interface. Impedance spectroscopy data is usually interpreted using a suitable equivalent circuit model of the electrochemical system, that is, the tissue-electrode interface. The impedance data would help examine a model of the interface to understand how the induced charge is transferred from the implanted electrode to the tissue, and how this would change over time [12]. Hence, the modeling of the interface can be considered as the first step towards investigating the changes in the tissue-electrode interface to propose solutions for mitigating degradation at the interface. In addition, an intricate analysis of the electrodes could help improve the efficiency of the charge transfer process and long term stability of the implants. “Ideal” conditions would ascertain an interface with moderately low impedance facilitating an efficient charge transfer across the interface. The voltage excursion induced by a stimulus should also maintain a reversible thermodynamic equilibrium well within the water window.

Our earlier biobotic demonstrations have used commercially available stainless steel wires as stimulation electrodes. While we have had success with these inexpensive and readily available electrodes, the stainless steel lacks the reversible redox reactions at the tissue electrode interface and the resulting higher charge injection capacity observed in conventional stimulation electrodes (e.g. iridium oxide). Moreover, stainless steel may be prone to corrosion and dissolution [13]. Such properties may lead to an unwarranted change in the impedance and likely a failure of the tissue-electrode interface. Relatively better alternative electrode materials could be gold or silver, which are often used for neural recording applications. These are relatively superior in quality and better conductors than stainless steel [14]. Silver has a slightly better conductivity than gold and silver-based electrodes have been recently and successfully used in biobotic applications [4,5]. While a direct comparison between silver and gold electrodes has not been made, gold with enhanced electrochemical properties can prove to be a more favorable alternative. Electrodeposition of conductive polymer PEDOT:PSS–poly(3,4-ethylene-dioxythiophene) polystyrene sulfonate–over gold provides electrochemical enhancement resulting with a high charge injection capacity [15,16]. This is due to dopants introducing extra mobile charge carriers and decreased interface impedance [15,17]. Gold with such enhancement has improved redox reactions making it more suitable as stimulation electrodes [15]. Gold is also one of the most common metals used in microfabrication of electrodes. For antennal stimulation of insects, we are limited by the electrode location. Hence, microfabrication further helps to meet design and dimensional constraints to satisfy implantation requirements within the narrow space of the roach antenna for biobotic applications.

As another alternative option, we also investigated the potential of liquid metal-based electrodes in biobotic applications [18]. Eutectic gallium-indium (EGaIn) is a low toxicity liquid metal alloy [19,20]. Despite having low viscosity, this liquid metal is quasi-stable because of a thin oxide layer on the surface [20–22]. Owing to the liquid state of the metal, an EGaIn electrode can be easily made to have various resistance values just by changing the dimensions of its contact area or diameter of the containing tube. This means it is also possible to adjust tissue-electrode impedance to desired values by changing the amount of EGaIn and/or combining it with various materials including hydrogels or even other electrode materials like stainless steel. EGaIn has also been demonstrated to be a non-toxic neural electrode by successful targeted stimulation of neurons in microfluidic cell culture platforms [20].

This paper is a first-ever attempt to directly compare the electrochemical performance of these novel electrode types for a neurostimulation application. This study focuses on *in vitro* comparison per se as *in vivo* studies also introduce other variabilities due to biological factors and requires a more complicated biobotic behaviour output evaluation to compare different electrode types. Such an *in vivo* comparison would be the next and future stage of this study. We have also kept a more detailed electrochemical analysis of the redox reactions underlying these results for a future publication.

This paper has been divided into the following sections: Section II details the preparation methods for the tested biobotic electrodes: (i) commercially available stainless steel wire electrodes (SS), (ii) micro fabricated gold electrodes (Au) with PEDOT:PSS electrochemical enhancement (AuP), (iii) eutectic gallium-indium alloy-based liquid metal electrodes (EGaIn), and (iv) a hybrid electrode with EGaIn coating on stainless steel wires (SS-EGaIn). Section III describes the experimental methods for analysing and characterizing the electrodes through a number of *in vitro* tests: (a) electrochemical impedance spectroscopy to provide an equivalent circuit model for the interface, (b) cyclic voltammetry to determine charge injection capacity of the electrodes, and (c) accelerated aging of the electrodes. Section IV presents the obtained results with their discussion followed by the conclusion in Section V.

## Materials and methods

### Biobotic electrodes

We prepared and tested five different types of electrodes *in vitro* for this work: (i) SS–electrodes used in our biobotic experiments–served as a baseline. (ii) AuP–micro fabricated and electrochemically enhanced–as an alternative to SS; Au is typically a favorable material for recording electrodes and we electrochemically enhance the Au electrodes to AuP with higher charge injection capacity, aiming to make them suitable as stimulation electrodes. (iii) EGaIn–tested to investigate properties of liquid metal electrodes with tunable resistance. (iv) “Hybrid” SS-EGaIn–provided means to modify properties of SS with EGaIn or vice versa.

#### (i) SS electrodes

**Fig 2:**
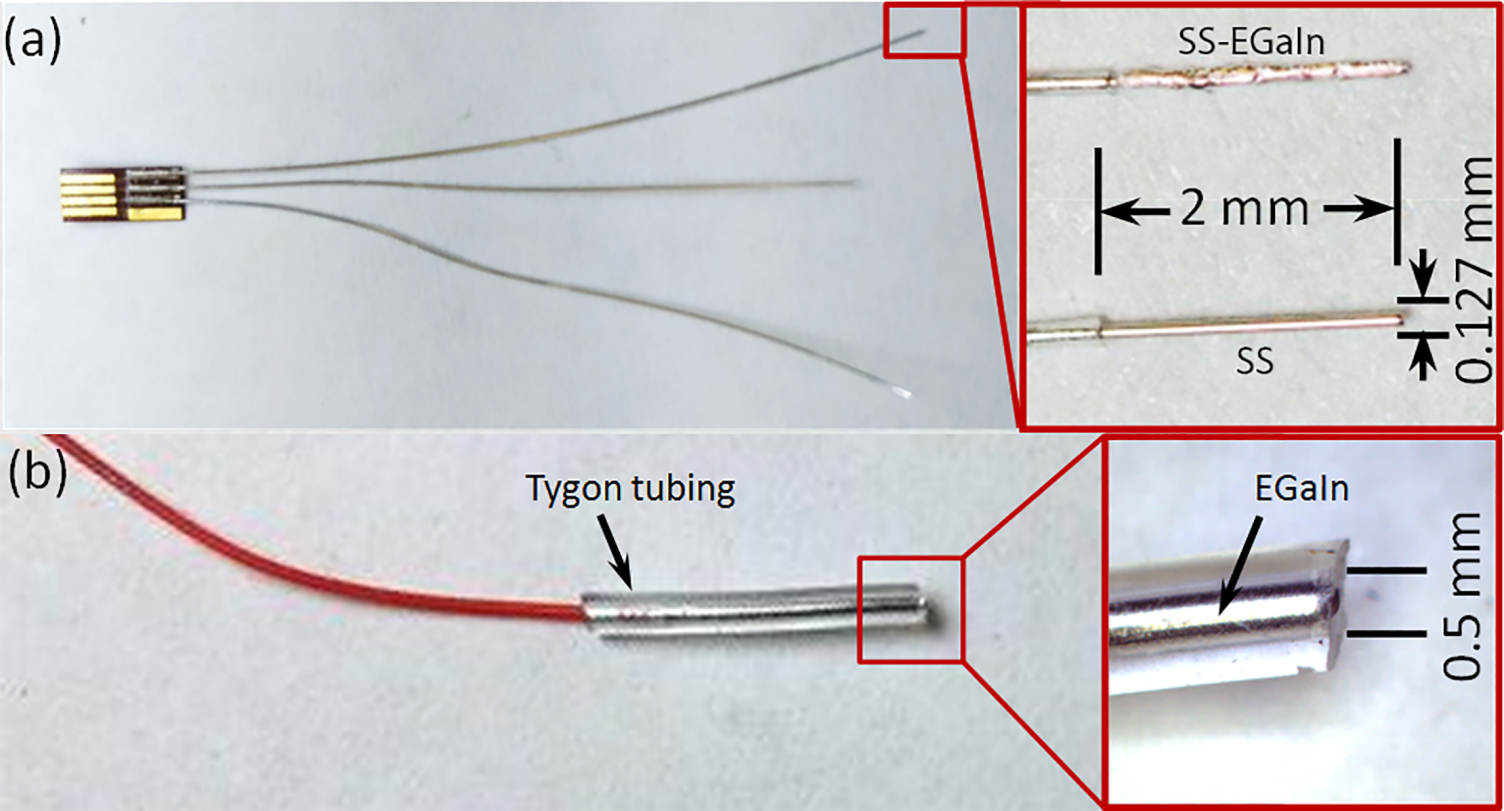
Stainless steel and EGaIn based electrodes. (a) Electrode setup with commercially available 127 μm PFA insulated SS wire electrodes. One end of the electrodes are soldered onto a small connector for connection to a neurostimulation backpack or an external circuit using an FFC connector. Insulation from the other ends are removed by 3.5 cm using a scalpel; these are implanted into the mesothorax or flagellum of an antenna of a cockroach. (inset) tip of SS and EGaIn coated SS electrodes. (b) EGaIn in 1.5 cm long Tygon tubes (0.5 mm inner diameter, 1.5 mm outer diameter) for use as an electrode. The tube is filled with EGaIn to a length of 1 cm with EGaIn from one end, carefully preventing any trapped air bubbles inside. This end formed the electrolyte-electrode interface with saline while a metal wire was inserted 0.5 mm deep from the other end for connection to external circuit.

PFA insulated 127 μm stainless steel wire electrodes (A-M Systems) have been used for biobotic experiments in the past (Fig 2) [3]. These commercially available, biocompatible, single strand wire electrodes require minimal preparation and allow easy implantation in the insects. The surgical implantation process involves inserting about 3.5 cm long wires into the flagellum of each antennae of an anesthetized cockroach as working electrodes–left electrode and right electrode–and into the mesothorax as a common or counter electrode [3]. The other ends of the three wire electrodes are soldered onto a small connector for connection to a neurostimulation backpack via a flexible flat cable (FFC) connector. In this work, we used the same type of stainless steel wires, with 2 mm of insulation removed at the tips.

#### (ii) Microfabricated AuP electrodes

**Fig 3:**
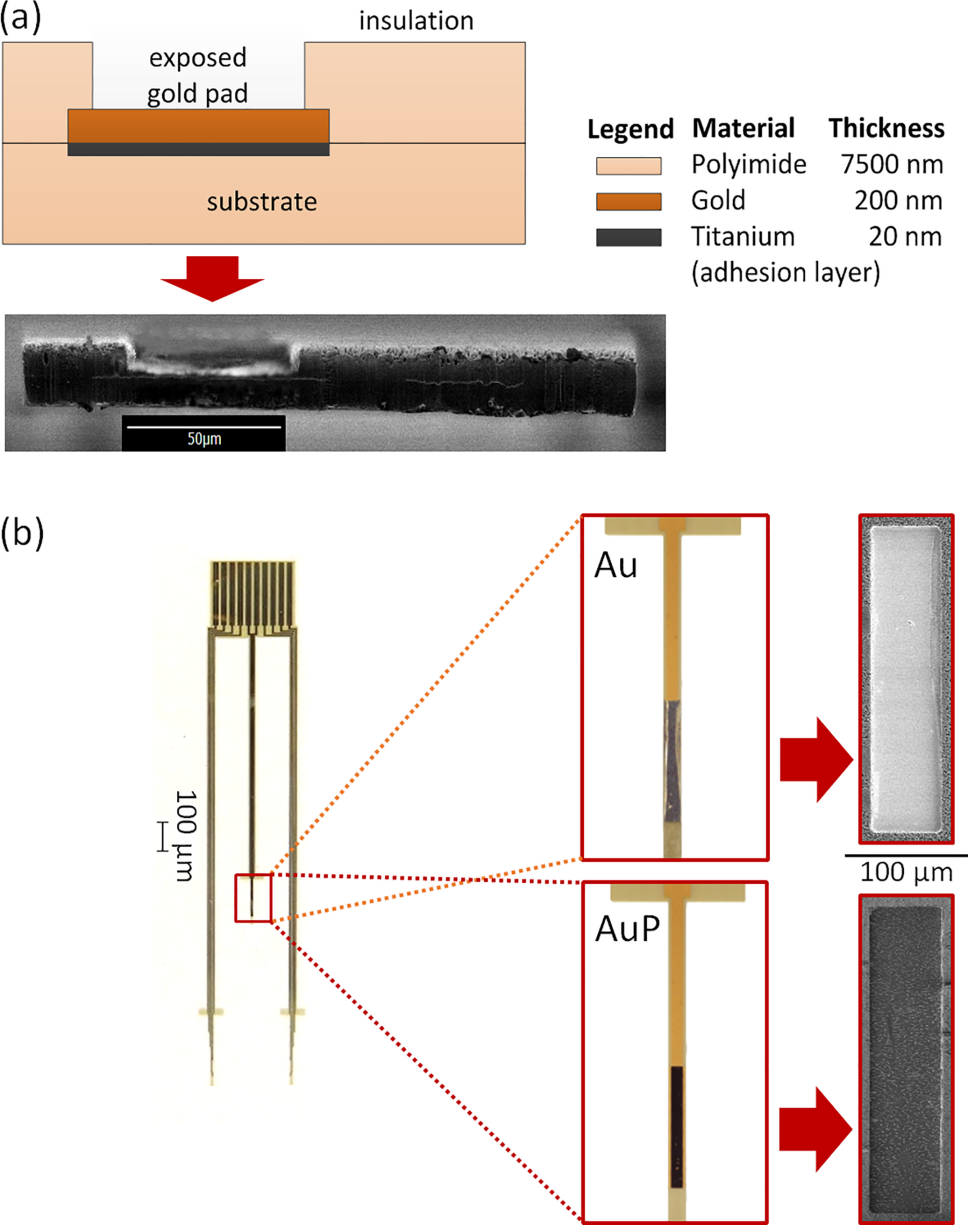
Micro fabricated gold electrodes. (a) Cross-sectional illustration (top) and SEM image (bottom) of a microfabricated gold electrode. The two thin horizontal lines running along the SEM image are the gold layers. (b) Microfabricated electrodes used in the experiments (left). The flat top end is inserted to an FFC connector for for connection to external circuit. (inset) Optical microscope and SEM images show an electrode pad at the electrode tip before and after electropolymerization using PEDOT:PSS conductive polymer.

We designed and microfabricated gold electrodes (Fig 3) to have three strands, similar to a complete stainless steel electrode. The center strand is the common electrode while the outer two are the working electrodes for antennal implantation. The electrode pad on the center strand measures 127 μm × 1270 μm. Each of the antennal strands have pads measuring 51 μm × 737 μm along the length of the strand. Gold traces interconnect these exposed electrode pads to the larger connector pads at the other end of the flexible electrode. These pads were designed to match FFC connectors to connect to external circuits.

We microfabricated these electrodes on a silicon carrier wafer. A sacrificial metal layer (Cr/Cu/Cr) was first deposited on the silicon wafer. Subsequently, a 7.5 μm layer of non-photodefinable polyimide was spin-coated onto the silicon substrate and cured. Metal deposition was used to deposit a 15 nm titanium adhesion layer followed by a 150 nm gold layer using a DC sputtering system. The metal was patterned using wet etching techniques. A subsequent 7.5 μm thick insulating polyimide layer was deposited and cured on top. Both polyimide layers were deposited using spin coating, and cured in an oven at 325°C for 1 hour. An aluminum hard mask was deposited by thermal evaporation, and the electrodes were patterned using reactive ion etching (80% O_2_ and 20% CF_4_). The electrodes were then peeled from the silicon substrate for release. The sacrificial metal layer was etched using wet etching techniques to release the devices from the carrier wafer.

Subsequently, we electro-polymerized PEDOT-PSS over the gold stimulation pads. Electrode pad contact surface modification by depositing PEDOT:PSS conductive polymer is known to enhance electrochemical properties in terms of increasing the charge injection capacity and decreasing the impedance of the tissue-electrode interface [15,16,23]. We prepared PEDOT: PSS solution in the laboratory by stirring 35 mg of 3,4-ethylenedioxythiophene and 250 mg poly(styrenesulfonic acid sodium salt) in 25 ml of deionized water for 2 h using a magnetic stirrer [15]. For electropolymerization of the electrodes, we found that a potentiostatic approach produced more consistent results than a galvanostatic one. A voltage was applied to maintain 100 μA/mm^2^ for 5 minutes. We determined this duration empirically by optimizing the achieved deposition results. The electrodes for electrochemical polymerization were immersed in the PEDOT:PSS solution with a 254 μm × 76.20 mm platinum wire (A-M Systems) as the counter electrode. After the electropolymerization process, the deposited layer of PEDOT:PSS was checked for uniformity using an optical microscope. Based on microscopic observations and pre-deposition profilometer measurements, the thickness of the PEDOT:PSS polymer layer are estimated to be around 7 μm. Fig 3 shows sample electro-polymerized pads with characteristic black coloration of PEDOT:PSS coating. We found electropolymerization of PEDOT:PSS on stainless steel to be difficult. The results were not convincing and hence we only used PEDOT:PSS with the microfabricated gold electrodes.

#### (iii) EGaIn electrodes

An EGaIn electrode is usually formed with liquid metal inside a capillary or tube. A simple approach would be to inject EGaIn through the tube using a syringe, and to make interfaces with a cell or tissue medium at one end of the tube and a solid metal wire for external circuit connections at the other. We used 1.5 cm long Tygon tubes (0.5 mm inner diameter, 1.5 mm outer diameter) and filled the length of 1 cm with EGaIn from one end, carefully preventing any trapped air bubbles inside. This end formed the electrolyte-electrode interface with saline while a wire was inserted 0.5 mm deep from the other end for external connection. Fig 2 shows a typical EGaIn-in-tube electrode. Our interest was in electrochemically observing how EGaIn itself would perform as a stimulation electrode.

#### (iv) SS-EGaIn electrodes

We also used a hybrid EGaIn-stainless steel electrode (Fig 2), which seemed to be more practical for roach implantations compared to the EGaIn-in-tube electrodes. For this, we dipped the tip of SS electrodes in EGaIn 20 times in quick succession. In these electrodes, EGaIn formed an intermediary liquid-solid metal interface between the SS electrodes and surrounding electrolytic medium.

### Experimental methods

We used a benchtop potentiostat (Gamry Reference 600) to run electrochemical impedance spectroscopy (EIS) and cyclic voltammetry (CV) tests in a 0.9% sodium chloride saline solution at room temperature in a 30 ml closed-top glass jar. We used both 3- and 2-electrode electrochemical cells. We simulated the tissue-electrode interface with a saline solution based electrolyte-electrode interface. The reference electrode was a 4.8 mm × 4.8 mm silver-silver chloride (Ag-AgCl) electrode and a 254 μm × 76.20 mm platinum wire acted as the counter electrode for 3-cell measurements (both electrodes from A-M Systems). The measurements in a 3-cell set up minimizes the effect of the electrolytic resistance and is expected not to be affected by the redox reaction at the counter electrode. In contrast, 2-cell measurements may be influenced by these, but may simplify EIS measurements for an *in vivo* setup with roach-bots, thanks to simpler implantation requirements. We have analyzed and compared data from 2-cell and 3-cell measurements to validate accuracy and justify the use of 2-cell over 3-cell measurements.

#### (a) Electrochemical impedance spectroscopy

EIS expresses a Bode plot with magnitude and phase of measured impedance over the frequency range. A 10 mV AC voltage (typical EIS small-signal value) with zero DC bias voltage was used as the input signal for the EIS measurements. The interface impedance was measured between 100 Hz and 100 kHz at 10 discrete frequencies per decade.

**Fig 4:**
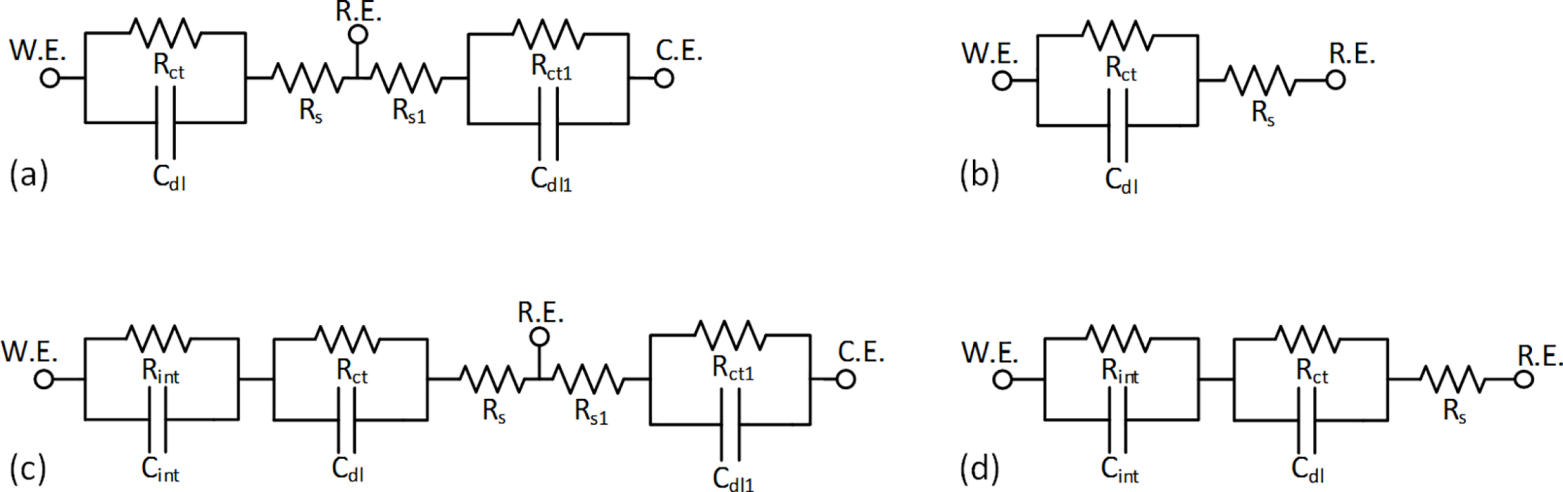
Equivalent circuit model of an electrolyte-electrode interface. (a, b) Model of an interface between working and reference electrodes labeled as W.E. and R.E. respectively, shown in a 3-cell circuit (a) with a counter electrode C.E. and in a 2-cell circuit (b). The charge conducting interface is conceived of a parallel RC network with parameters *R*_*ct*_ (the charge transfer resistance) and *C*_*dl*_ (the double layer capacitance). *R*_*s*_ is the electrolytic resistance. (c, d) Modified version of the equivalent circuit model in 4(a, b) for SS-EGaIn electrodes. An additional RC parallel network is considered for the intermediate layer between SS and EGaIn, where network parameters *R*_*int*_ and *C*_*int*_ function corresponding to *R*_*ct*_ and *C*_*dl*_ respectively.

A Bode plot usually indicates an RC circuit impedance of the interface equivalent circuit model decreasing with frequency. Applied stimuli causes a voltage drop across this RC circuit network which constitutes the electrolyte-electrode interface and the electrolytic resistance. The charge conducting interface is conceived of a parallel RC network with parameters *R*_*ct*_ (the charge transfer resistance), and *C*_*dl*_ (the double layer capacitance) responsible for the charge transfer across the interface. A lower *R*_*ct*_ and a higher *C*_*dl*_ would ensure improved charge transfer across the interface. *R*_*s*_ is the electrolytic resistance. These model parameter values help in understanding the behavior of the interface, and hence, the properties of the electrode in question. Fig 4 shows the interface model with working, reference, and counter electrodes labeled as W.E., R.E., and C.E. respectively.

For analyzing the EIS data, we iteratively estimated values of the circuit parameters from the EIS using Gamry Echem Analyst software. The estimated values can also be used to compute the interface impedance, and vice versa, with Eqs (1)–(4) [24]:

$$\begin{array}{l} Z\left(\omega \right)=R_{s}+\frac{R_{ct}}{1+j\omega R_{ct}C_{dl}}\\ \;=R_{s1}+\frac{R_{ct}}{1+{\left(\omega R_{ct}C_{dl}\right)}^{2}}-j\frac{\omega R_{ct}{}^{2}C_{dl}}{1+{\left(\omega R_{ct}C_{dl}\right)}^{2}} \end{array}$$ (1)

$$Z\left(\omega \right)=Z^{\prime }\left(\omega \right)+jZ^{\prime \prime }\left(\omega \right),$$ (2)

where *ω* = angular frequency, *Z*′ = real part of the impedance, *Z*″ = imaginary part of the impedance, which in turn can be used to calculate [24],

$$R_{ct}\left(\omega \right)=\frac{{Z^{\prime \prime }}^{2}+{\left(Z^{\prime }-R_{s}\right)}^{2}}{Z^{\prime }-R_{s}},$$ (3)

$$\mathrm{and}\;C_{dl}\left(\omega \right)=-\frac{Z^{\prime \prime }}{\omega \left[{\left(Z^{\prime }-R_{s}\right)}^{2}+{Z^{\prime \prime }}^{2}\right]}.$$ (4)

We have used this model for the SS, Au, AuP, and EGaIn electrodes. We considered an additional RC parallel network for the intermediate layer between SS and EGaIn in the SS-EGaIn electrodes; *R*_*int*_ and *C*_*int*_ form the second network corresponding to *R*_*ct*_ and *C*_*dl*_ respectively (Fig 4).

#### (b) Cyclic voltammetry

We performed a linear sweep at a scan rate of 500 mV/s between ± 600 mV, thereby keeping measurements between the water window. At the working electrode, each cycle in a CV curve denotes a redox reaction indicated by the associated Faradaic current peaks. The area under the CV curve corresponds to the charge injection capacity.

#### (c) Accelerated aging of electrodes

In the scope of a biobotic study, we are generally interested in average biobotic operation duration of at least a week following electrode insertion. After the working time, the insects would be retired in an insect terrarium. To that effect, we performed *in vitro* accelerated aging of the electrodes at an elevated temperature to emulate the effect of real time aging in a relatively shorter period of time. The goal was to determine if an electrode and, by extension, the interface endured the saline environment for an extended period of time without adversely affecting the electrolytic medium or itself.

We elevated the temperature of our saline jar setup to 60°C using a digital stirring hotplate. The saline was stirred at a constantly slow rate and the saline level was replenished to its initial level with deionized water, as needed. The temperature was kept constant throughout the experiments and did not exceed 60°C to prevent non-linearity in the reaction rate [25]. We calculated experimental duration of about 26 h for a simulated real age of two weeks using Eq (5) [26],

$$Age_{T_{ref}}=Age_{T_{ET}}\times n^{\frac{\left[T_{ET}-T_{ref}\right]}{\theta }},$$ (5)

where $Age_{T_{ref}}$ = real time age at reference (or room) temperature *T*_*ref*_, $Age_{T_{ET}}$ = accelerated age at elevated temperature *T*_*ET*_, *θ* = temperature increment, and *n* = reaction rate = 2 for *θ* = 10°C [26,27].

We analyzed the aging effects on the electrode material using electrochemical analysis and microscopic imaging.

## Results and discussion

The outliers in each sample of the collected electrode data were eliminated by analysing the impedance values over the frequency range. This resulted in a refined sample size for each electrode type (n = 20). Then, the mean ± standard error at each frequency point per electrode type was calculated and graphically analysed.

### EIS

**Fig 5:**
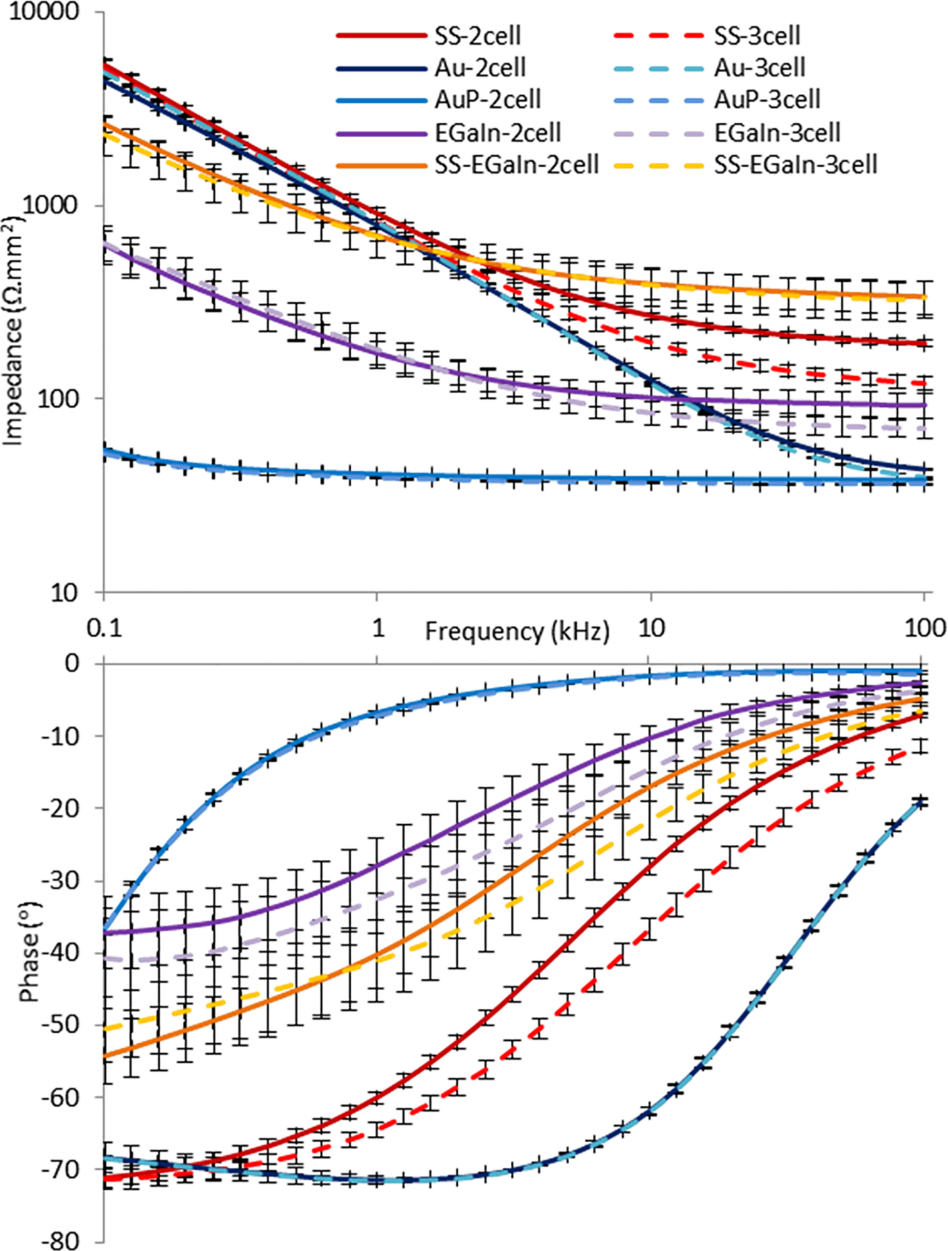
EIS plots for all electrodes types. Plots using 2-cell and 3-cell data for the same type of electrode have close match between impedances at corresponding frequencies. SS electrodes have high impedances, followed by Au electrodes comparable to SS at lower frequencies. EGaIn electrodes have low impedances with AuP the lowest among the tested electrodes. SS-EGaIn, a hybrid, formed by the addition of EGaIn to SS represents an enhancement of the SS electrodes.

**Fig 6:**
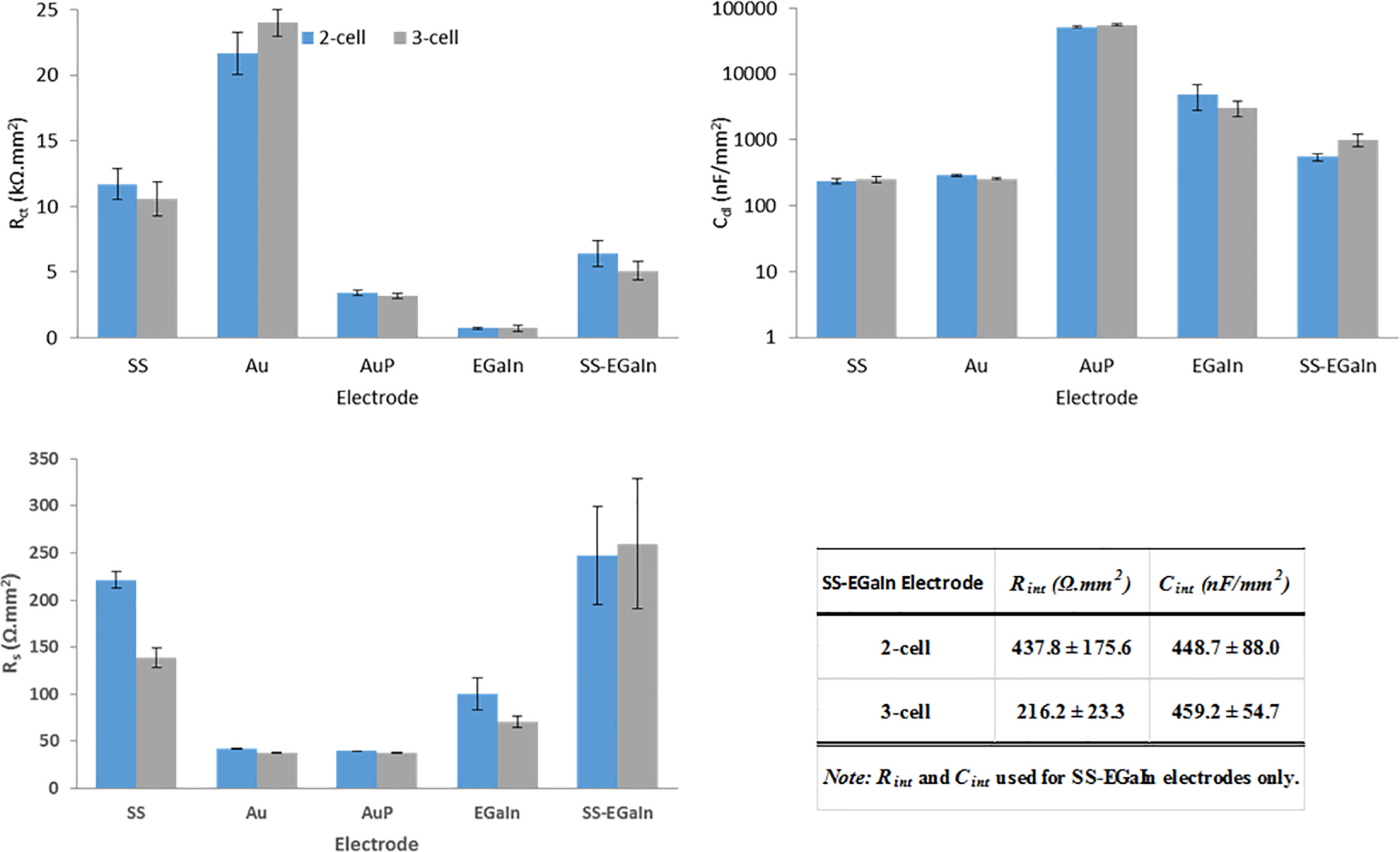
Mean values of interface circuit model parameters estimated from 2-cell and 3-cell measurements. (a) *R*_*ct*_−charge transfer resistance, (b) *C*_*dl*_−double layer capacitance, (c) *R*_*s*_−electrolytic resistance, and (d) *R*_*int*_ and *C*_*int*_−charge transfer parameters at the intermediate layer between SS and EGaIn in the SS-EGaIn electrodes. AuP and EGaIn electrodes have lower *R*_*ct*_ and higher *C*_*dl*_ values designating them the most favorable among the tested electrodes. Model parameters estimated from 2-cell and 3-cell data of the same electrode types have approximately equal values.

First, we examined the deviation between 2-cell and 3-cell measurements by analysing the EIS data (Fig 5) through the interface equivalent circuit model parameters (Figs 4 and 6). We also used paired sample *t*-test to study the impedance plots, using sets of impedance data at multiple frequency points (Table 1). For the *t*-test, we compared impedance at discrete frequency points with α = 0.05. In some cases, this comparison shows discrepancies between compared data as expected, most significantly for AuP. Nevertheless, our results did show a close match between impedances at corresponding frequencies and approximately equal values of the model parameters for the same type of electrodes used. In the constraints of this study, these results indicate that 2-cell measurements can effectively be used as an estimate, albeit not a direct measurement, for 3-cell measurements without compromising data accuracies. Therefore, for practical purposes, lesser number of surgical implantations could be used for an *in vivo* interface characterization setup with roach-bots in the future. The *in vivo* study of the tissue-electrode interface is underway and beyond the scope of this paper due to the introduced complexity caused by biological invariances.

**Table 1:** Paired sample *t*-test of 2-cell and 3-cell measurement of electrodes.

Electrode	*100 Hz*	*1 kHz*	*10 kHz*	*100 kHz*
SS—2-cell	*p>α*	*p>α*	*p<α*	*p<α*
SS—3-cell				
Au—2-cell	*p<α*	*p>α*	*p>α*	*p<α*
Au—3-cell				
AuP—2-cell	*p<α*	*p<α*	*p<α*	*p<α*
AuP—3-cell				
EGaIn—2-cell	*p>α*	*p>α*	*p>α*	*p>α*
EGaIn—3-cell				
SS-EGaIn—2-cell	*p>α*	*p>α*	*p>α*	*p>α*
SS-EGaIn—3-cell				

Note: α = 0.05.

Next, we objectively compared the different types of electrodes used in this study. While most biobotic experiments ran with SS electrodes in the past and provided satisfactory behavioural results, we have found that SS electrodes have mediocre electrochemical performance that could cause performance degradation in the long-term. As expected, SS electrodes have impedances higher than most other materials we used, with Au electrodes closely matching these at lower frequencies (Figs 5 and 6). This is justified by both electrodes having similar *R*_*ct*_ values, while a higher *C*_*dl*_ for Au manifests itself as relatively lower impedances at higher frequencies. The Au electrodes underwent significant improvements by the electropolymerization with PEDOT:PSS. This resulted in added charge carriers, and the effect was demonstrated by AuP electrodes having the lowest *R*_*ct*_ and the highest *C*_*dl*_ values among the tested electrodes, thereby suggesting a potentially good charge transfer across an interface upon stimulation. This corresponds to a decreased impedance, and a phase plot moving closer to zero and greater in value compared to the other electrodes, as shown in Fig 5. The second lowest impedance curve was obtained with EGaIn electrodes, a novel electrode material evaluated for neurostimulation in roach-bots here, with lower *R*_*ct*_, but *C*_*dl*_ not as high as that of AuP electrodes. Addition of EGaIn to SS (SS-EGaIn) gave a hybrid of the two electrodes and represents an enhancement of SS electrodes. While EGaIn demonstrated a more favorable performance compared to SS-EGaIn, the tests on SS-EGaIn allowed us to recognize a way to enhance the properties of SS electrodes and demonstrate the efficacy of this modification process.

We used a modified RC parallel network for the equivalent circuit model of SS-EGaIn to accommodate the introduced interface layer between SS and solution through EGaIn with the corresponding model parameters, *R*_*int*_ and *C*_*int*_. This extra layer of EGaIn on the surface of SS exhibited an improved charge transfer across the electrolyte-electrode interface.

As an exercise, we also used a more realistic equivalent circuit model of the interface by replacing the all the capacitances (*C*_*dl*_ and *C*_*int*_) with a constant phase element (*CPE*_*dl*_ and *CPE*_*int*_ respectively) to account for the nonuniformity in the surface structures. CPE impedance is defined as *(Y*_*0*_*(jω)*^*n*^*)*^*−1*^ for *CPE*_*dl*_ and *(Y*_*0*,*int*_*(jω)*^*n*,*int*^*)*^*−1*^ for *CPE*_*int*_. Tables 2 and 3 compares the results for both models, with the capacitive and CPE elements. Both models verify the results obtained in Fig 6 and confirm our analysis.

**Table 2:** Interface circuit model parameters–ideal interface.

Electrode	*R*_*int*_ *(Ω*.*mm*^*2*^*)*	*C*_*int*_ *(nF/mm*^*2*^*)*	*R*_*ct*_ *(kΩ*.*mm*^*2*^*)*	*C*_*dl*_ *(nF/mm*^*2*^*)*	*R*_*s*_ *(Ω*.*mm*^*2*^*)*
SS—2-cell	-	-	11.7 ±1.2	232.7 ±20.3	221.4 ±8.4
SS—3-cell	-	-	10.6 ±1.3	253.9 ±27.7	138.6 ±10.3
Au—2-cell	-	-	21.7 ±1.6	287.3 ±14.7	41.7 ±0.5
Au—3-cell	-	-	24.0 ±1.0	256.2 ±6.7	37.7 ±0.1
AuP—2-cell	-	-	3.4 ±0.2	51677.4 ±1265.1	39.5 ±0.3
AuP—3-cell	-	-	3.2 ±0.2	55106.8 ±2141.4	37.5 ±0.3
EGaIn—2-cell	-	-	0.7 ±0.1	4800.6 ±1769.8	100.3 ±15.2
EGaIn—3-cell	-	-	0.7 ±0.2	3014.0 ±783.8	70.3 ±6.2
SS-EGaIn—2-cell	437.8 ±175.6	448.7 ±88.0	6.4 ±1.0	550.5 ±72.7	247.3 ±51. 9
SS-EGaIn—3-cell	216.2 ±23.3	459.2 ±54.7	5.1 ±0.7	998. 5 ±221.4	259.8 ±69.0

*Note*: *R*_*int*_ and *C*_*int*_ used for SS-EGaIn electrodes only.

**Table 3:** Interface circuit model parameters–non-ideal interface.

Electrode	*R*_*int*_ *(Ω*.*mm*^*2*^*)*	*Y*_*0*,*int*_ *(×10*^*−9*^*)*	*n*,*int*	*R*_*ct*_ *(kΩ*.*mm*^*2*^*)*	*Y*_*0*_ *(×10*^*−9*^*)*	*n*	*R*_*s*_ *(Ω*.*mm*^*2*^*)*
SS—2-cell	-	-	-	14.4 ±1.0	733.0 ±80.2	0.86 ±0.01	202.6 ±7.7
SS—3-cell	-	-	-	14.8 ±1.1	997.0 ±147.9	0.84 ±0.01	122.7 ±9.9
Au—2-cell	-	-	-	28.9 ±5.9	1298.6 ±153.2	0.80 ±0.01	34.8 ±1.0
Au—3-cell	-	-	-	26.5 ±1.8	1516.8 ±185.5	0.79 ±0.01	32.2 ±1.1
AuP—2-cell	-	-	-	3.1 ±0.4	63041.7 ±2667.3	0.96 ±0.01	38.8 ±0.3
AuP—3-cell	-	-	-	3.3 ±0.4	72525.1 ±7113.2	0.96 ±0.01	37.6 ±0.4
EGaIn—2-cell	-	-	-	0.8 ±0.1	27091.7 ±13893.0	0.79 ±0.03	96.1 ±13.3
EGaIn—3-cell	-	-	-	0.9 ±0.2	26134.7 ±8226.9	0.79 ±0.03	62.5 ±5.4
SS-EGaIn—2-cell	237.9 ±43.1	36190.5 ±17448.7	0.70 ±0.03	6.8 ±1.1	6125.2 ±4239.2	0.84 ±0.02	252.9 ±71.0
SS-EGaIn—3-cell	228.9 ±31.3	74151.3 ±39991.9	0.70 ±0.08	5.6 ±0.8	10519.1 ±3621.0	0.83 ±0.04	143.7 ±29.1

*Note*: *R*_*int*_, *Y*_*0*,*int*_, and *n*_*int*_ used for SS-EGaIn electrodes only.

### CV to determine charge injection capacity

**Fig 7:**
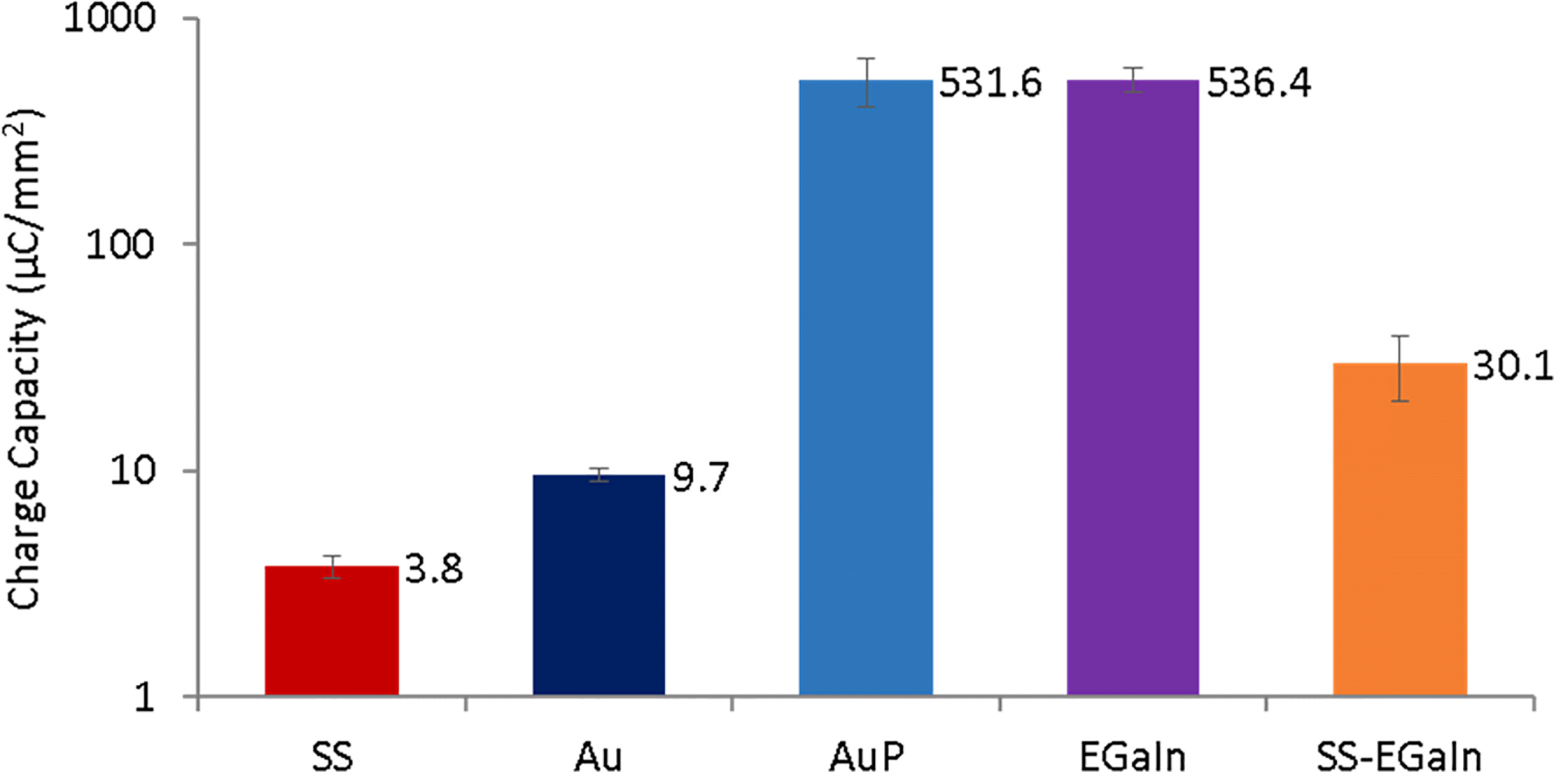
Charge injection capacity for all electrodes types in 2-electrode cells. The charge injection capacity of all electrode types are determined from CV plots and were found to be correlated to the corresponding EIS impedance. AuP and EGaIn electrodes have the highest charge capacity, with AuP having a prominent redox reaction.

To further reinforce findings from EIS data, we analyzed CV curves to determine the charge injection capacity of the electrodes (Fig 7, S1 Fig). We have performed preliminary analysis of the CV and accelerated aging data for both 2- and 3-electrode cells, but charge capacity data for 2-cell only were presented and discussed here owing to their similarity with 3-cell data. From the CV, we determined the charge injection capacity of all electrode types and verified their correlation with the corresponding EIS impedance. SS electrodes had the lowest capacity, but demonstrated a distinct redox reaction. Electropolymerization of Au leading to AuP electrodes resulted in relatively prominent redox reactions and a near 50 times improvement in charge capacity relative to Au alone. This could be explained as an effect of the PEDOT:PSS introducing extra mobile charge carriers and facilitating faster ion transport across the polymer coating [15–17]. EGaIn electrodes had a charge capacity similar to AuP. Consequently, SS-EGaIn had increased charge capacity compared to SS electrodes. One characteristic we observed with EGaIn-based electrodes was the dominance of the series resistance that introduced a sharp slope to the curve.

### Accelerated aging

**Fig 8:**
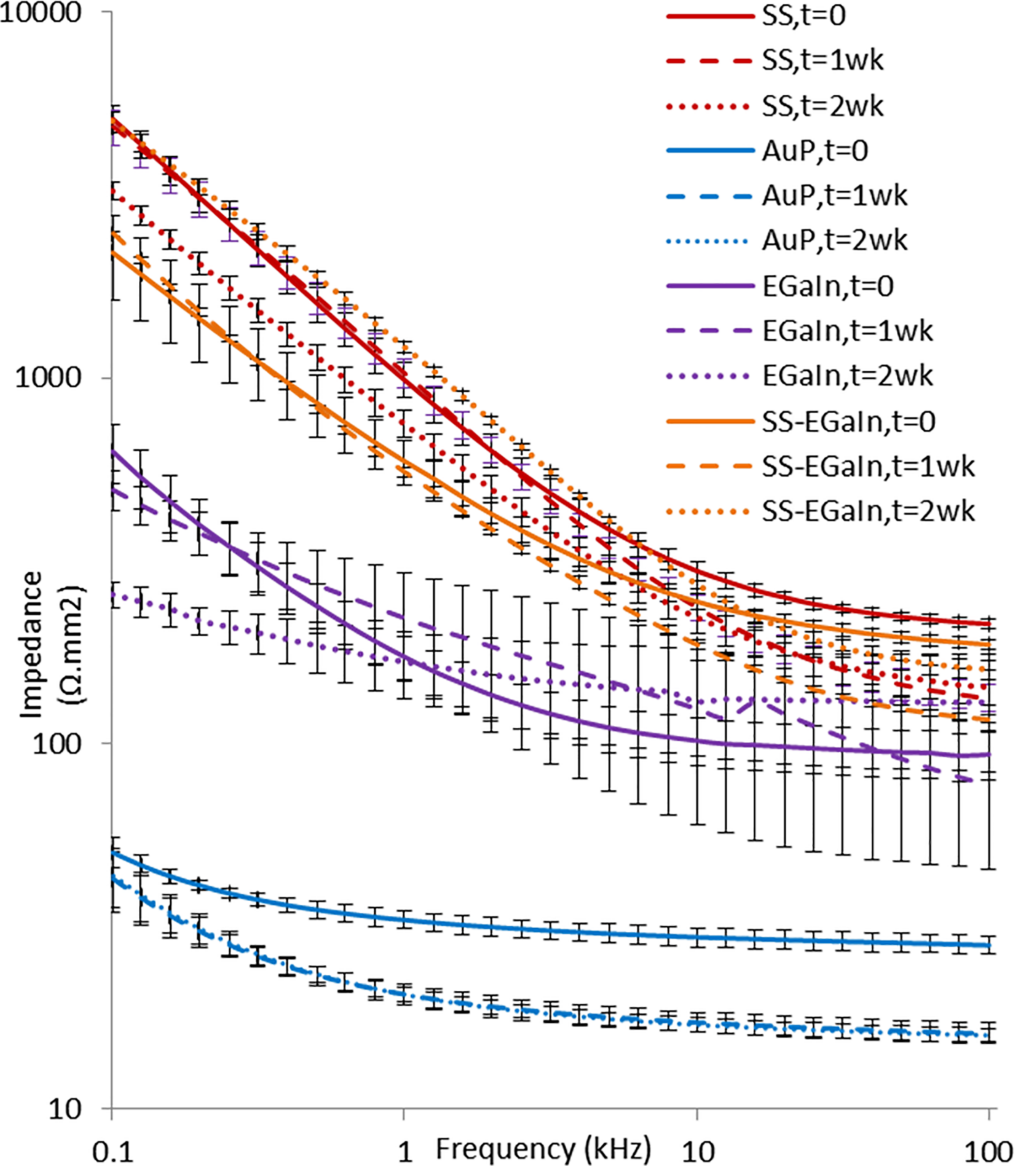
EIS plots of electrodes obtained over a period of two weeks. Results for 2-electrode cell data are shown at t = 0, 1 week, and 2 weeks. After two weeks, moderate changes in the impedance plots were observed, owing to the salt accumulation at the interfaces. EGaIn started to display some irregularity due to the changes on its oxide layer brought upon by interaction with water molecules. SS-EGaIn began behaving more like SS electrodes after two weeks due to disassociation of EGaIn from the surface of the SS electrodes.

**Fig 9:**
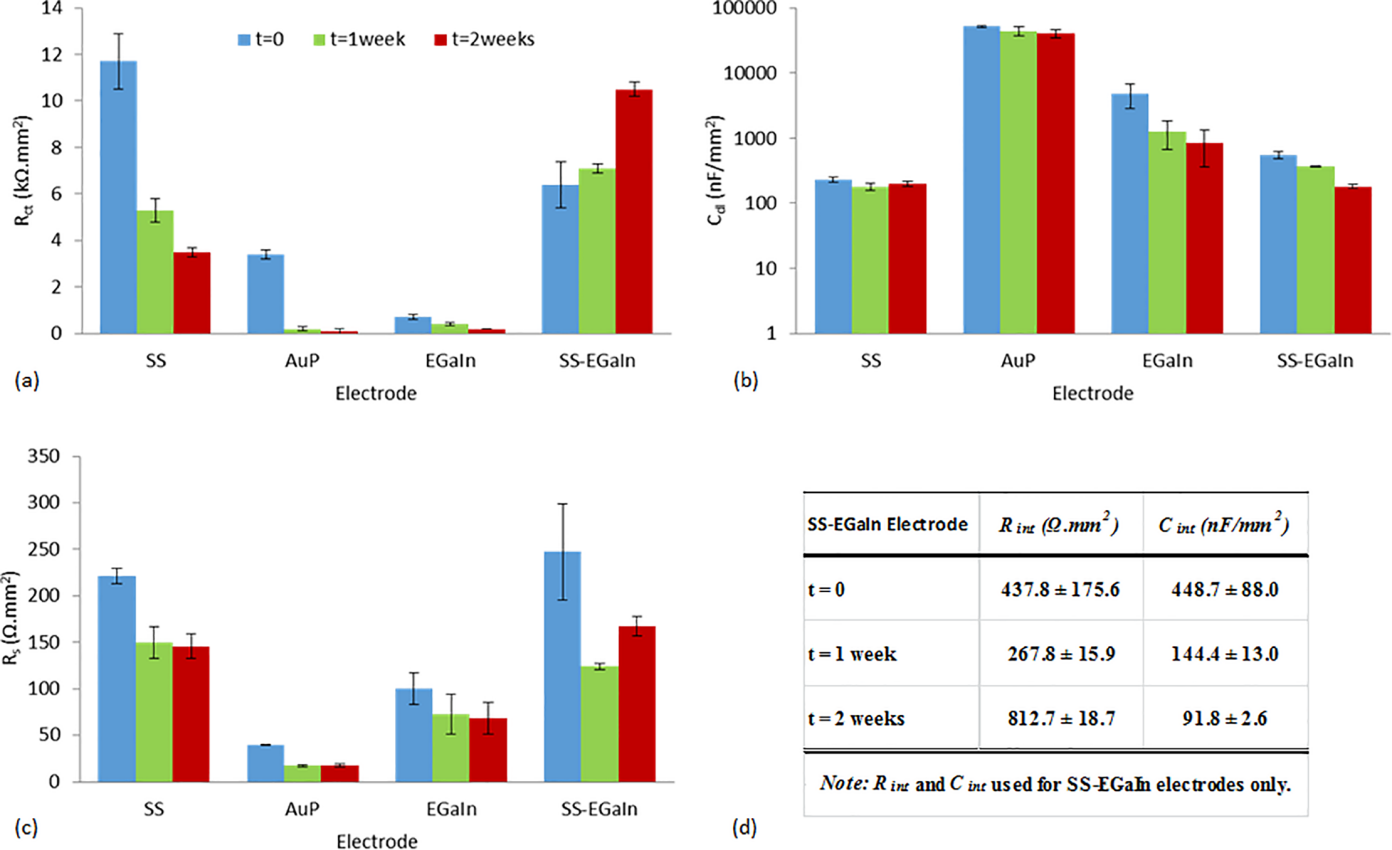
Mean change in 2-cell circuit model parameters with time. (a) *R*_*ct*_−charge transfer resistance, (b) *C*_*dl*_−double layer capacitance, (c) *R*_*s*_−electrolytic resistance, and (d) *R*_*int*_ and *C*_*int*_−charge transfer parameters at the intermediate layer between SS and EGaIn in the SS-EGaIn electrodes. Changes in the parameter values reflect the trends observed in the EIS plots. *R*_*ct*_ changed for all electrodes except for EGaIn whose *C*_*dl*_ decreased by about five times over the period of two weeks. The *C*_*dl*_ of SS stayed fairly constant, while that of AuP changed, but remained considerably high. Parameters of the SS-EGaIn electrodes were found to gradually become similar to the initial parameters of the SS electrodes.

We performed accelerated aging tests with SS, AuP, EGaIn and SS-EGaIn electrodes, with a sample size of n = 4 per electrode type (Figs 8 and 9). We noticed moderate changes in the overall impedance plot of the electrodes during the two week simulated period, but observed some decrement potentially due to salt accumulation at the interfaces by time. We also noticed higher deviation and some unpredictability in the EGaIn electrodes with aging. While *R*_*ct*_ remained similar, *C*_*dl*_ decreased by about five times over the period of two weeks. This may be due to the effect of water on the oxide layer. The interaction of water molecules with oxide has been shown to cause it to get thicker over time, changing the composition of the oxide layer to a gelatinous complex of gallium oxide monohydroxides (GaOOH), although this is a slow process [28]. In principle, it may be possible to use electrochemical reduction to help remove the oxide on the liquid metal *in vitro* although this may be challenging to implement *in vivo* [29].

**Fig 10:**
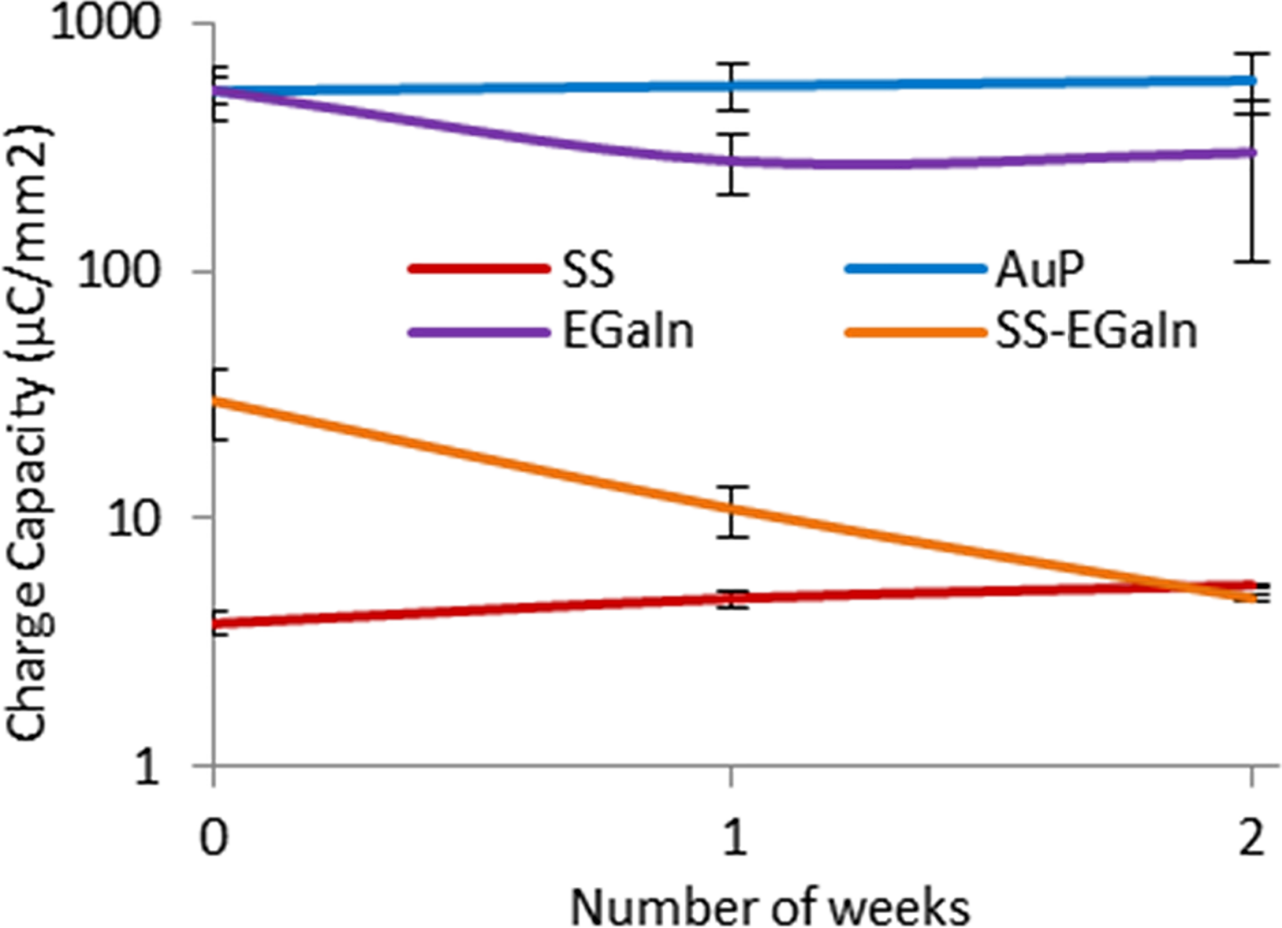
Charge injection capacity of electrodes calculated from accelerated aging data. The AuP electrodes were found to have the highest charge injection capacity which appeared consistent throughout the two weeks duration. It was followed by EGaIn electrodes which had the second highest charge capacity, and SS-EGaIn and SS electrodes which had relatively low capacity with SS electrodes having the lowest capacity. SS-EGaIn had increased charge injection capacity compared to SS, but it dropped and became more like that of SS with time as the EGaIn dislodged from the SS surface.

We also noticed some major changes in the parameters corresponding to the SS-EGaIn electrodes, and the rise in impedance making them similar to the SS electrodes suggesting a short longevity of the SS-EGaIn electrodes. Upon checking the electrodes using an optical microscope, we noticed some discoloration (possibly due to oxidation) and cracking in the EGaIn coating after two weeks. In addition to the changing chemical composition of the oxide layer on the EGaIn surface, the dissociation of the EGaIn from the SS surface could explain the rise in the values of the interface resistances. Dissociation of the EGaIn possibly resulted in the increased resistance at the intermediate layer between the SS and EGaIn, while salt accumulation could have caused the dip in the impedance value halfway through the aging. On the other hand, neither SS nor AuP electrodes had a noticeable change in appearance. Moreover, the charge capacity of AuP and SS electrodes stayed reasonably constant with regards to the standard error (Fig 10). The *C*_*dl*_ of SS also stayed fairly constant, while that of AuP changed, but remained considerably high.

## Conclusions

This paper presented a comparative study of five types of electrodes for neurostimulation of biobots. The *in vitro* analysis presented in this paper is an essential work for direct electrochemical comparison and lays the foundation for a future *in vivo* analysis of these electrodes. Due to the possible variations in the biological factors as well as the need for a more complicated analysis of how each electrode type perform towards inducing certain biobotic behavior, we decided to dedicate this study to the test of *in vitro* factors only. We used *in vitro* electrochemical analysis of the electrode-electrolyte interface to assess and compare the performance of stainless steel, PEDOT-PSS coated gold and EGaIn electrodes. We have also tested the coating of stainless steel with EGaIn as a hybrid solution. From the EIS data, we have determined that 2- and 3-electrode cell data can lead to similar comparison results, thereby allowing future *in vivo* electrode characterization with biobots using 2-electrode cells only. This simplifies the surgical procedure for such future studies. The accelerated aging of the tested electrodes verified the one to two weeks reliable operation of the electrodes while letting us speculate about the potential failure modes. While we have observed most electrodes to retain some level of initial characteristics, EGaIn and SS-EGaIn had a declining performance during the accelerated aging process. SS-EGaIn electrodes were superior to SS electrodes for about a week, but gradually became similar to SS in two weeks’ time. Therefore, SS-EGaIn electrodes may be preferred as neurostimulation electrodes for experiments lasting about a week. Taking the overall data for impedance, interface model parameters and charge injection capacity into consideration, PEDOT-PSS coated gold electrodes (AuP) maintained a consistently superior performance during the test period. Hence, AuP electrodes can be considered as an improved alternative to SS electrodes.

## Supporting information

S1 Fig Cyclic voltammograms and charge injection capacity for all electrodes types in 2-electrode cells. (ZIP)
