# Supplementary material for: In vitro electrochemical assessment of electrodes for neurostimulation in roach biobots
Source: PLoS One. 2018 Oct 10;13(10):e0203880. doi: 10.1371/journal.pone.0203880 (PMC6179205; doi:10.1371/journal.pone.0203880)
Supplement: S1 Fig — (ZIP) [file pone.0203880.s001.zip › Supporting Information.docx]

***In vitro* Electrochemical Assessment of Electrodes for Neurostimulation in Roach Biobots**

Tahmid Latif^1^, Michael McKnight^1^, Michael D. Dickey^2^, and Alper Bozkurt^1^

^1^Department of Electrical and Computer Engineering, North Carolina State University, Raleigh, NC 27695-7911, USA

^2^Department of Chemical and Biomolecular Engineering, North Carolina State University, Raleigh, NC 27695-7905, USA

Corresponding author: Alper Bozkurt, e-mail: aybozkur@ncsu.edu

**Supporting Information**


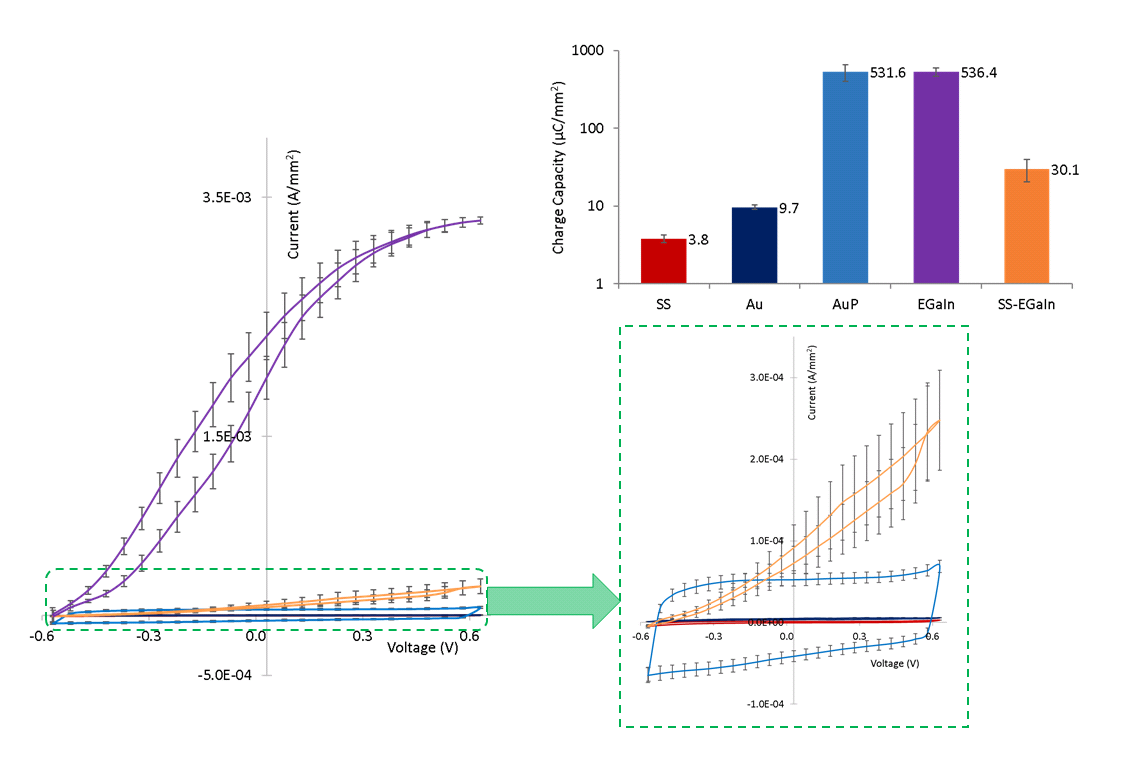


**S1 Fig.** Cyclic voltammograms and charge injection capacity for all electrodes types in 2-electrode cells.
