# Supplementary figures and images for: In vitro electrochemical assessment of electrodes for neurostimulation in roach biobots
Source: PLoS One. 2018 Oct 10;13(10):e0203880. doi: 10.1371/journal.pone.0203880 (PMC6179205; doi:10.1371/journal.pone.0203880)

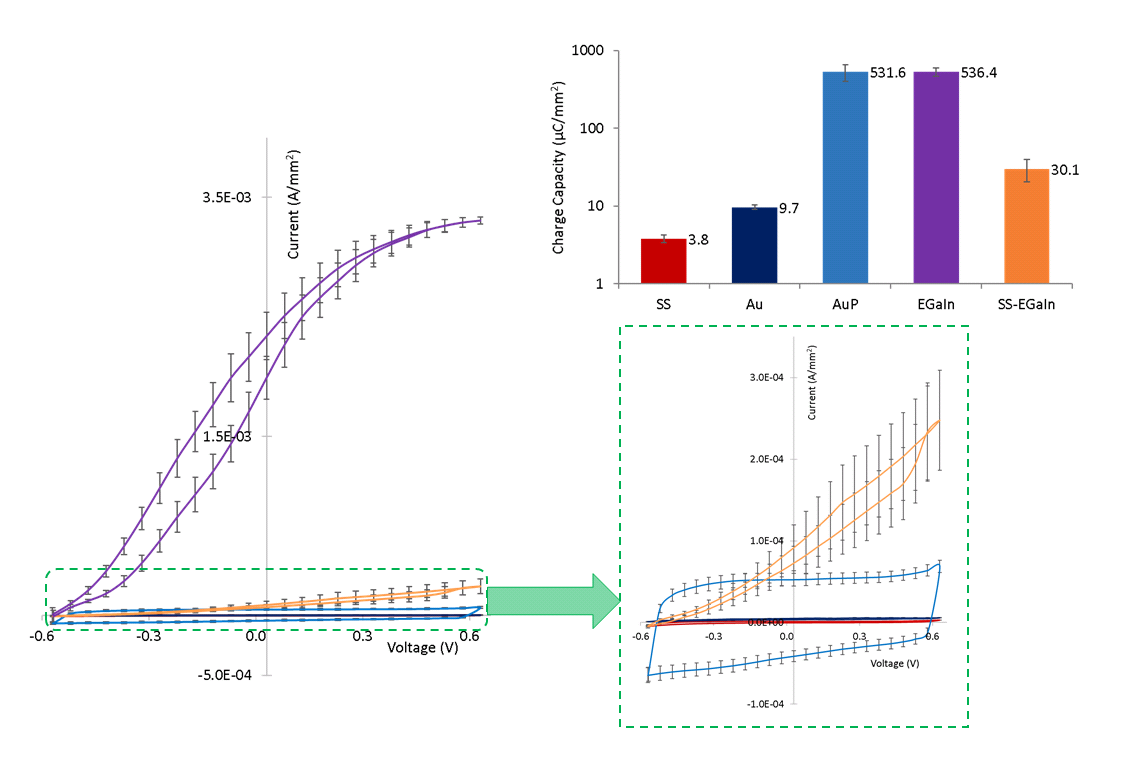

Supplement: S1 Fig — (ZIP) [file pone.0203880.s001.zip › S1 Fig.tif]
